# Management of Large Tongue Schwannoma – A Short Report

**Published:** 2016-03

**Authors:** Jayanta Medhi, Hanifa Akhtar Laskar, Deepanava Jyoti Das, N Brian Shunyu, Ankit Jitani, Vandana Raphael, Rajni Thabah

**Affiliations:** 1 *Department of ENT and Head and Neck Surgery, North-eastern Indira Gandhi Regional Institute of Health and Medical Sciences.*; 2 *Department Of Pathology, North-eastern Indira Gandhi Regional Institute of Health and Medical Sciences.*; 3 *Department Of Anesthesia, North-eastern Indira Gandhi Regional Institute of Health and Medical Sciences.*

Schwannoma is a benign nerve sheath tumor composed of schwann cells. Oral cavity is a rare site for schwannomas, tongue being the most common location. Here we are presenting a case of a young adult who presented with a huge swelling in the tongue which was removed by mandibulotomy approach and pre-operative tracheostomy. 

A 22-year-old male patient presented to the outpatient department with a history of swelling in the tongue for the last 4 years with progressive difficulty in swallowing food and change of voice over the last few months. Upon examination a large swelling was observed on the posterior part of the tongue compromising the oropharyngeal inlet. The approximate size of the swelling was 5cmx4cm. After proper clinical evaluation the patient was advised to obtain a magnetic resonance imaging (MRI) study of the oral cavity, which showed it to be a nerve sheath tumor (Schwannoma) originating from the hypoglossal nerve branch. The patient was admitted for surgery. As difficult intubation was anticipated, pre-operative tracheostomy was performed.

The tongue mass was approached by right paramedian mandibulotomy using a transcervical lip split incision. Post operative histopathological examination of the removed specimen showed hypercellular ‘Antony A’ area with plump spindle cells and hypocellular ‘Antony B’ area in a Hematoxylin & eosin stain (200x). This confirmed the diagnosis for a schwannoma.

As each and every case is unique in its presentation, so is the management. The idea of presenting the above case is to emphasise the role of selection for the proper approach and foresee the preventable complications while working around the airway. 

**Fig 1:**
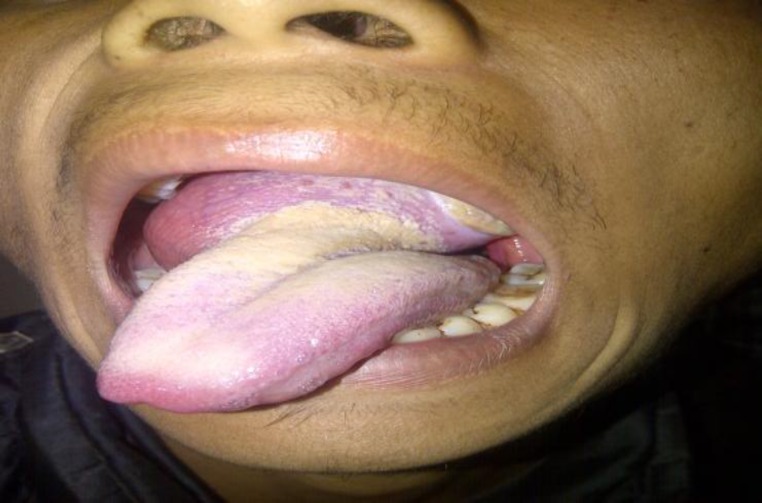
Pre-operative appearance of the tongue mass

**Fig-2:**
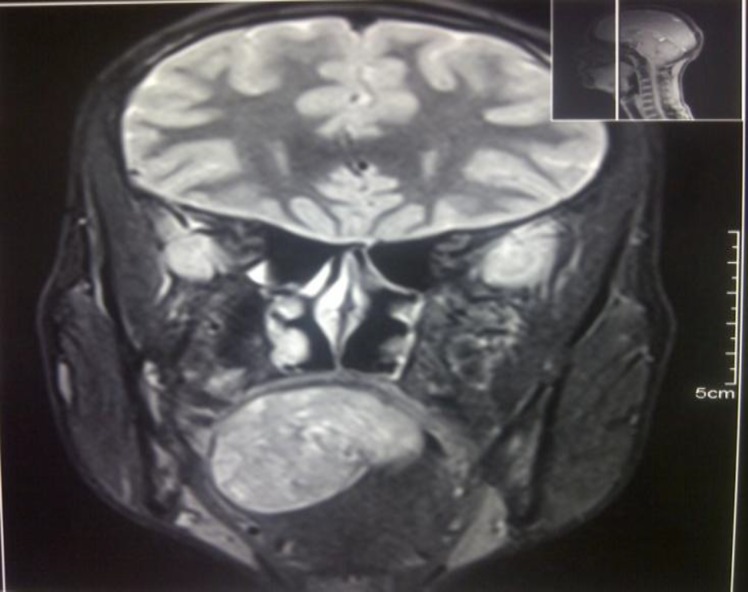
MRI study of the oral cavity showing the tongue lesion

.

